# FastPop: a rapid principal component derived method to infer intercontinental ancestry using genetic data

**DOI:** 10.1186/s12859-016-0965-1

**Published:** 2016-03-09

**Authors:** Yafang Li, Jinyoung Byun, Guoshuai Cai, Xiangjun Xiao, Younghun Han, Olivier Cornelis, James E. Dinulos, Joe Dennis, Douglas Easton, Ivan Gorlov, Michael F. Seldin, Christopher I. Amos

**Affiliations:** Department of Biomedical Data Science, Dartmouth College, Hanover, NH USA; Department of Genetics, Dartmouth College, Hanover, NH USA; Centre for Cancer Genetic Epidemiology, Cambridge University, Cambridge, UK; Rowe Program in Genetics, U.C. Davis, Davis, CA USA

**Keywords:** Population structure, Principal component, Ancestry, Genome-wide association study

## Abstract

**Background:**

Identifying subpopulations within a study and inferring intercontinental ancestry of the samples are important steps in genome wide association studies. Two software packages are widely used in analysis of substructure: Structure and Eigenstrat. Structure assigns each individual to a population by using a Bayesian method with multiple tuning parameters. It requires considerable computational time when dealing with thousands of samples and lacks the ability to create scores that could be used as covariates. Eigenstrat uses a principal component analysis method to model all sources of sampling variation. However, it does not readily provide information directly relevant to ancestral origin; the eigenvectors generated by Eigenstrat are sample specific and thus cannot be generalized to other individuals.

**Results:**

We developed FastPop, an efficient R package that fills the gap between Structure and Eigenstrat. It can: 1, generate PCA scores that identify ancestral origins and can be used for multiple studies; 2, infer ancestry information for data arising from two or more intercontinental origins. We demonstrate the use of FastPop using 2318 SNP markers selected from the genome based on high variability among European, Asian and West African (African) populations. We conducted an analysis of 505 Hapmap samples with European, African or Asian ancestry along with 19661 additional samples of unknown ancestry. The results from FastPop are highly consistent with those obtained by Structure across the 19661 samples we studied. The correlations of the results between FastPop and Structure are 0.99, 0.97 and 0.99 for European, African and Asian ancestry scores, respectively. Compared with Structure, FastPop is more efficient as it finished ancestry inference for 19661 samples in 16 min compared with 21–24 h required by Structure. FastPop also provided scores based on SNP weights so the scores of reference population can be applied to other studies provided the same set of markers are used. We also present application of the method for studying four continental populations (European, Asian, African, and Native American).

**Conclusions:**

We developed an algorithm that can infer ancestries on data involving two or more intercontinental origins. It is efficient for analyzing large datasets. Additionally the PCA derived scores can be applied to multiple data sets to ensure the same ancestry analysis is applied to all studies.

**Electronic supplementary material:**

The online version of this article (doi:10.1186/s12859-016-0965-1) contains supplementary material, which is available to authorized users.

## Background

Genome wide association (GWA) studies usually evaluate data from thousands of individuals. Identifying the subpopulations within the data set and inferring biogeographic origins of the samples are important steps in the conduct of any study. Not allowing for population substructure in the analysis will introduce false positives [1]. Furthermore, one usual step in quality control procedures checks for Hardy-Weinberg equilibrium, often just in the controls. If the population being studied comprises two or more subpopulations, Hardy-Weinberg equilibrium will be violated for any SNPs with variability in allele frequencies among the subsets. Two software packages are widely used in analysis of substructure: Structure and Eigenstrat. Structure assumes each individual may inherit a proportion of its ancestry from multiple distinct populations and then estimates an ancestry proportion for each subpopulation [2, 3]. The setup of running Structure is complex as it requires tuning multiple parameters. Also when large samples are involved, Structure requires considerable computational time. Eigenstrat, implementing the program smartPCA, uses principal component analysis (PCA) to model ancestry variation among the samples [4–6]. PCA has been a standard procedure in population genetics studies for over 30 years. The continental origin variations in allele frequencies among individuals can be elaborated in a lower dimensional space using the derived eigenvectors to score individuals. However, PCA does not fulfill the requirement of ancestry inference as it does not estimate the proportional ancestry origin of each individual. Furthermore the current implementation of Eigenstrat returns eigenvectors for a specific population that cannot be generalized to another sample. To extend the use of PCA in association analysis and develop a fast and accurate method for ancestry inference, we have developed FastPop, an R package that allows users to estimate the proportion of intercontinental ancestry for each individual. Furthermore, the scores derived from PCA analysis in FastPop can be generalized to other studies provided the same set of markers are used. Human population history tends to follow gradients of gene flow [7], and we have incorporated flow among major populations to assist in assignment of major ancestral origins of participants.

## Implementation

### Principal components analysis

We selected 2318 SNPs across the whole genome based on having a large fixation index (FST) value among European, African and Asian populations for PCA analysis. We conducted PCA analysis of 505 Hapmap samples with European, African or Asian ancestry along with a collection of 19661 additional samples of unknown ancestry. To perform PCA, we use the eigendecomposition method, which parses the covariance relationships among markers.

Define $$ {\boldsymbol{X}}_{N\times P}=\left(\begin{array}{ccc}\hfill {x}_{11}\hfill & \hfill \cdots \hfill & \hfill {x}_{1P}\hfill \\ {}\hfill \vdots \hfill & \hfill \ddots \hfill & \hfill \vdots \hfill \\ {}\hfill {x}_{N1}\hfill & \hfill \cdots \hfill & \hfill {x}_{NP}\hfill \end{array}\right), $$ where N and P are the number of samples and SNPs, respectively.

For the mean-centered data matrix, we need to compute the mean on each SNP,

$$ {\overline{x}}_{.j}=\frac{{\displaystyle {\sum}_{i=1}^N}{x}_{ij}}{N},\  where\ i=1,\cdots, N\  and\ j=1,\cdots, P. $$

Then, we generate the mean-centered data matrix as

$$ {\boldsymbol{Y}}_{N\times P} \equiv \left[\begin{array}{ccc}\hfill {x}_{11}-{\overline{x}}_{.1}\hfill & \hfill \cdots \hfill & \hfill {x}_{1P}-{\overline{x}}_{.P}\hfill \\ {}\hfill \vdots \hfill & \hfill \ddots \hfill & \hfill \vdots \hfill \\ {}\hfill {x}_{N1}-{\overline{x}}_{.1}\hfill & \hfill \cdots \hfill & \hfill {x}_{NP}-{\overline{x}}_{.P}\hfill \end{array}\right]. $$

Assume that there are larger number of samples than number of SNPs (*N* > *P*). We construct the covariance matrix as

$$ {\boldsymbol{C}}_{P\times P} = \frac{1}{N-1} \cdot {\boldsymbol{Y}}^T\boldsymbol{Y}\ . $$

Since the covariance matrix **C** is symmetric and positive definite, the eigenvalues of **C** are real and positive semi-definite. The eigendecomposition of covariance matrix **C** can be applied to calculate the eigenvalues *λ*_*i*_ and the eigenvectors ***v***_*i*_ of **C** satisfying that

$$ \boldsymbol{C}{\underline {\boldsymbol{v}}}_i={\lambda}_i{\underline {\boldsymbol{v}}}_i. $$

which can be written in matrix form as ***CV*** = ***V*Λ**,

where $$ {\boldsymbol{\Lambda}}_{P\times P}=\left[\begin{array}{ccc}\hfill \begin{array}{ccc}\hfill \begin{array}{c}\hfill {\lambda}_1\hfill \\ {}\hfill 0\hfill \end{array}\hfill & \hfill \begin{array}{c}\hfill 0\hfill \\ {}\hfill {\lambda}_2\hfill \end{array}\hfill & \hfill \begin{array}{c}\hfill 0\hfill \\ {}\hfill 0\hfill \end{array}\hfill \end{array}\hfill & \hfill \cdots \hfill & \hfill \begin{array}{c}\hfill 0\hfill \\ {}\hfill 0\hfill \end{array}\hfill \\ {}\hfill \vdots \hfill & \hfill \ddots \hfill & \hfill \vdots \hfill \\ {}\hfill \begin{array}{ccc}\hfill 0\hfill & \hfill 0\hfill & \hfill 0\hfill \end{array}\hfill & \hfill \cdots \hfill & \hfill {\lambda}_P\hfill \end{array}\right] $$ is a diagonal matrix with diagonal eigenvalues *λ*_*i*_.

In PCA, the *λ*_*i*_ in **Λ** are extracted according to size descent order (i.e. *λ*_1_ ≥ *λ*_2_ ≥ ⋯ ≥ *λ*_*P*_) and the matrix $$ {\boldsymbol{V}}_{P\times P}=\left(\begin{array}{cc}\hfill \begin{array}{cc}\hfill {\underline{v}}_1\hfill & \hfill {\underline{v}}_2\hfill \end{array}\hfill & \hfill \begin{array}{cc}\hfill \cdots \hfill & \hfill {\underline{v}}_P\hfill \end{array}\hfill \end{array}\right) $$ consists of eigenvectors corresponding to *λ*_*i*_.

For our purposes we find that selection of SNPs to maximize interancestral variability ensures that the first few eigenvectors capture interpopulation variation. We therefore obtain a low dimensional projection, score matrix ***Z***_*N* × *k*_ = ***Y***_*N* × *P*_ × ***P***_*P* × *k*_ (1). Where k = 2 or 3 is adequate for capturing ethnic similarities when considering 3 or 4 continental origins respectively. The eigenvectors are

$$ {\boldsymbol{P}}_{P\times k} = \left(\begin{array}{cc}\hfill \begin{array}{cc}\hfill {\underline{v}}_1\hfill & \hfill {\underline{v}}_2\hfill \end{array}\hfill & \hfill \begin{array}{cc}\hfill \cdots \hfill & \hfill {\underline{v}}_k\hfill \end{array}\hfill \end{array}\right)\ . $$

Once we select the first k eigenvectors, named as SNP weights, which we would like to keep among the principal components computed from the discovery data, we can predict new scores in the new data using pre-computed SNP weights.

Let $$ {\boldsymbol{U}}_{M\times P}=\left(\begin{array}{ccc}\hfill {u}_{11}\hfill & \hfill \cdots \hfill & \hfill {u}_{1P}\hfill \\ {}\hfill \vdots \hfill & \hfill \ddots \hfill & \hfill \vdots \hfill \\ {}\hfill {u}_{M1}\hfill & \hfill \cdots \hfill & \hfill {u}_{MP}\hfill \end{array}\right) $$ be a new data with M samples and the same P SNPs as in the original analysis. Then, generate the mean-centered matrix, $$ {\boldsymbol{W}}_{N\times P} \equiv \left[\begin{array}{ccc}\hfill {u}_{11}-{\overline{u}}_{.1}\hfill & \hfill \cdots \hfill & \hfill {u}_{1P}-{\overline{u}}_{.P}\hfill \\ {}\hfill \vdots \hfill & \hfill \ddots \hfill & \hfill \vdots \hfill \\ {}\hfill {u}_{N1}-{\overline{u}}_{.1}\hfill & \hfill \cdots \hfill & \hfill {u}_{NP}-{\overline{u}}_{.P}\hfill \end{array}\right] $$.

Using the SNP weights ***P***_*P* × *k*_ from the original analysis, compute the new score matrix, Z*_N × *k*_ = ***W***_*N* × *P*_ × ***P***_*P* × *k*_ (2). For prediction of new score matrix, we recommend that the SNP weights should be generated from large samples (N> > P) to avoid variance shrinkage if the eigenvectors will be applied to a subsequent data set [8].

### Ancestry analysis

**Fig. 1:**
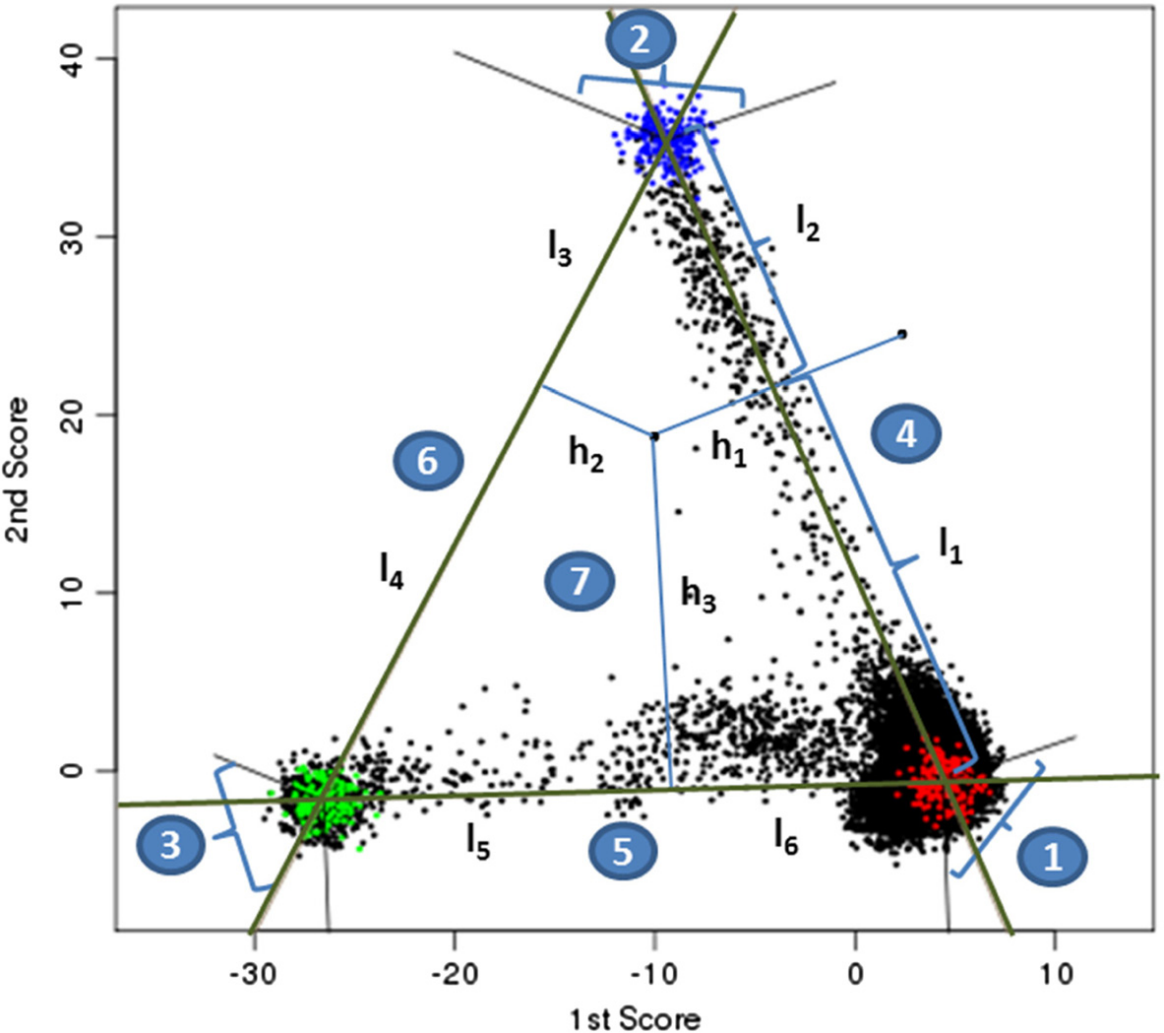
Proportion of ancestry inference using first and second PCA score. X and Y axis denote the first and second PCA score generated by FastPop. Red, blue, green and black denote Hapmap samples with European, Africana, Asian ancestry and studied samples with unknown ancestry. The centroids of each population were computed for Hapmap samples. Three lines in dark green were drawn connecting the centroids; six extra line perpendicular to triangle sides and across the centroids were drawn in grey. Individuals in area 1–3 are classified as pure European, Afrian and Asian origin; samples in area 4–6 have mixture origin of two adjacent populations; samples in area 7 have mixture origin of European, African and Asian ancestry. h1, h2 and h3 denote the distance between the samples in the triangle to the sides of the triangle; l1-l6 denote the distance between the image of the sample at the triangle sides and the population

We started with three well characterized continental populations, studied by the HapMap consortium of European, Asian, and African descent, since characterizing ancestry for these populations is often a major goal in genome-wide association studies. The centroid of each known Hapmap population was characterized by the multivariate mean of the first and second score (Fig. 1). A triangle reflecting the usual clines of intermarriage among continental groups was created by connecting the lines connecting the centroids (lines in dark green). Six lines perpendicular to the triangle sides and originating at the centroids were obtained (lines in grey). Individuals of unknown ancestry can be divided into three groups based on their location relative to the triangle: points in area 1–3 are classified as 100 % European, African or Asian ancestry, respectively; points in areas 4–6 have mixture origin of two adjacent populations; points in area 7 have a mixture origin of European, African and Asian ancestry. For points in area 4–6, the distance between the closest point on the triangle was identified and the admixture proportion is the proportional distance to each centroid. For example, for a sample in area 4, if the distances along the European-African line are L1 to the European and L2 to the African centroids, then the proportion of European ancestry for this individual is computed as $P=\frac{\frac{1}{L1}}{\frac{1}{L1}+\frac{1}{L2}}$ (3), the proportion of African ancestry is 1-P. For a point inside the triangle, let H1, H2 and H3 denote the distance between the point and the three sides of the triangle; L1-L6 denote the distance between the point and the nearest perpendicular projects onto the ancestry triangle. The proportion of European for this individual is then computed as

$P=\left(\frac{\frac{1}{H1}\times \frac{1}{L1}}{\frac{1}{L1}+\frac{1}{L2}}+\frac{\frac{1}{H3}\times \frac{1}{L6}}{\frac{1}{L6}+\frac{1}{L5}}\right)/\left(\frac{1}{H1}+\frac{1}{H2}+\frac{1}{H3}\right)$ (4), with similar calculations yielding the proportion of African and Asian ancestry. Hapmap samples can be downloaded from http://hapmap.ncbi.nlm.nih.gov/downloads/genotypes/hapmap3_r3/plink_format/.

The approach can also be generalized to include additional populations. When we plotted the first three PC scores for individuals coming from four distinct populations (European, Asian, African and Native American, along with a population of Mexican Americans of unknown ancestry), we observed a three-dimensional tetrahedron instead of a two dimensional triangle for three populations, which suggested we could extend the triangle algorithm to a tetrahedron-based algorithm to incorporate four populations. When applied to four populations, a similar approach to that taken for 3 populations is employed. We first define regions for which the closest point to an ancestry tetrahedron is a vertex, and for those individuals, a single continental ancestry is assigned. For points outside the tetrahedron but closest to a side, we project to the nearest face of the tetrahedron (as described in the Supplementary methods) and then use equation 4 to estimate the proportions of ancestry for each of three origins for that face. For points inside the tetrahedron, an extension of equation 4 to encompass a mixture of 4 populations is applied. In this case, the projections are to each of the four faces of the tetrahedron. For interior points, once the sample was mapped to the two-dimensional face (for example, the face formed by L1, L2 and L6 in Fig. 4), we applied equations 3 and 4 developed for three populations to estimate the proportions of European, Asian and Native American (denoted in red, green and purple) on this surface. We then performed the estimation of ancestry for each ancestry plane as indicated above for equations 3 and 4. For interior points the final estimated proportion of each ancestry is the average of the proportions from each of calculations.

### Software implementation

In order to use FastPop, users need to provide the input file in a correct format. FastPop takes the cleaned genotype file coded in additive model as the input file. The usual options for data cleaning includes removing individuals or SNPs with a high missing rate. We observed that an additional “population” may be identified when the missing rate for the samples was higher than 0.05. It is also critical that the SNP genotype data are in forward strand and it is easy to use PLINK to flip the alleles. We provided the reference allele file in the package for users to check the allele information.

**Fig. 2:**
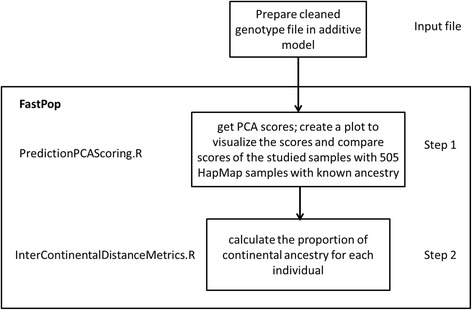
Flow chart of intercontinental ancestry analysis using FastPop

There are two steps using FastPop: first, FastPop will compute scores for individuals based on eigenvectors from PCA analysis; second, it will estimate the proportional ancestry of each individual based on the scores generated in first step (Fig. 2). FastPop is implemented in R programing language. The function “PredictionPCAScoring.R” enables the user to compute scores based on eigenvectors from PCA analysis given by 2318 SNP weights; create a plot to visualize the scores and compare scores of the studied samples with 505 HapMap samples with known ancestry. The function “InterContinentalDistanceMetrics” calculates the proportion of each continental ethnicity from scores of individuals and this function also works for inference using eigenvectors as input directly. The output file is ancestry.out which has six columns including sample ID, first PCA score, second PCA score, proportions of European, African and Asian ancestries. The whole package is freely available at https://sourceforge.net/projects/fastpop/files.

## Results

**Fig. 3:**
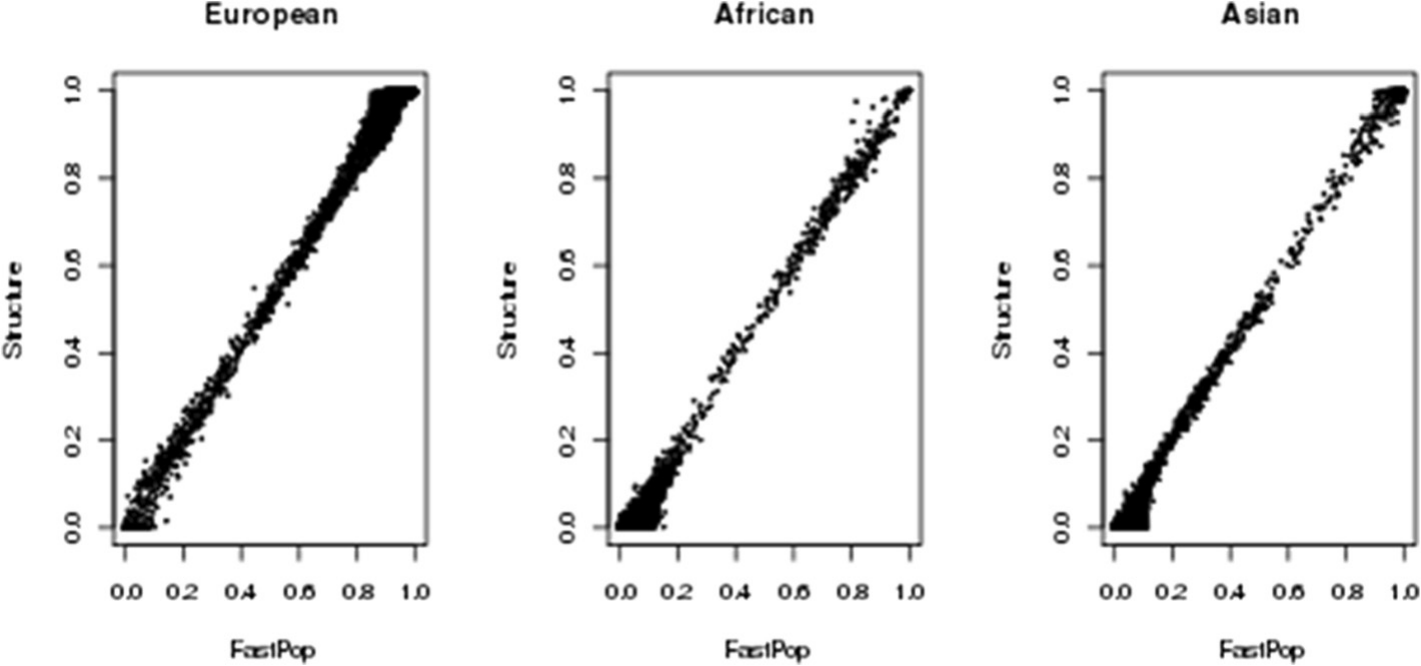
Comparison of estimated proportion of ancestry between FastPop and Structure for 19661 individuals. X and Y axees denote the proportion of ancestry for each individual from FastPop and Structure

We applied FastPop on intercontinental ancestry analysis of a data set including 19661 individuals from studies performed in the U.S. and Europe primarily of European descent, but with some additional self-reported African-American and Asian members. We used a large sample size to avoid any potential for overfitting and to ensure the intrasample variance is consistent for application to future studies. In order to evaluate the results from FastPop, we also ran Structure on the same set of data to benchmark our results. Structure was run without/with prior population information for Hapmap samples. For each individual, we compared the estimated proportions of European, African and Asian ancestry between FastPop and Structure. The results from FastPop are highly consistent with those found using Structure across the 19661 samples (Fig. 3). The correlations of the results between FastPop and Structure are 0.99, 0.97 and 0.99 for European, African and Asian, respectively. Further, we studied different cutoff values of estimated proportions of ancestry from 0.9 to 0.7 to assign the individuals to each continental ancestry, and FastPop had similar accuracy compared with Structure. For example, when we used a stringent cutoff value of 0.9 in both FastPop and Structure, >95 % of the individuals identified by Structure were also identified by FastPop. When the cutoff value was relaxed to 0.7, the concordance rate increased to 99.8 % (Table 1). While FastPop retains the same level of accuracy as Structure, it is much faster in performing the analysis compared with Structure. FastPop finished ancestry inference for 20166 samples in 16 min, while Structure required 21 h when implemented without prior ancestry information and 24 h when implemented with prior ancestry information for HapMap samples. Structure was run with parametric settings “BURNIN 10000 NUMREPS 1000 INFERALPHA 1 POPSPECIFICALMBDA 1”.

**Table 1:** Comparison of assigned ancestry using different cutoff value between FastPop and Structure

Cutoff value		CEU	YRI	CHB
	Without prior population information for Hapmap samples			
0.9	PCA Scores	17520/16329/16325	64/62/55	740/721/719
0.8	PCA Scores	18016/17928/17928	175/165/159	773/768/767
0.7	PCA Scores	18171/18122/18122	266/263/260	799/796/796
	With prior population information for Hapmap samples			
0.9	PCA Scores	17510/16329/16321	69/62/59	743/721/719
0.8	PCA Scores	18017/17928/17928	174/165/159	774/768/768
0.7	PCA Scores	18167/18122/18121	267/263/260	799/796/796

Number in each cell indicates: No. assigned by structure/No. assigned by FastPop/No. common in both methods. Structure analysis was conducted with/without prior population information for Hapmap samples. Structure was run was run under admixture model. Parameter setting “BURNIN 10000 NUMREPS 1000 INFERALPHA 1 POPSPECIFICALMBDA 1”. Without prior population information, the running time is 21:16:00 and 23:30:00 for with prior population information

**Fig. 4:**
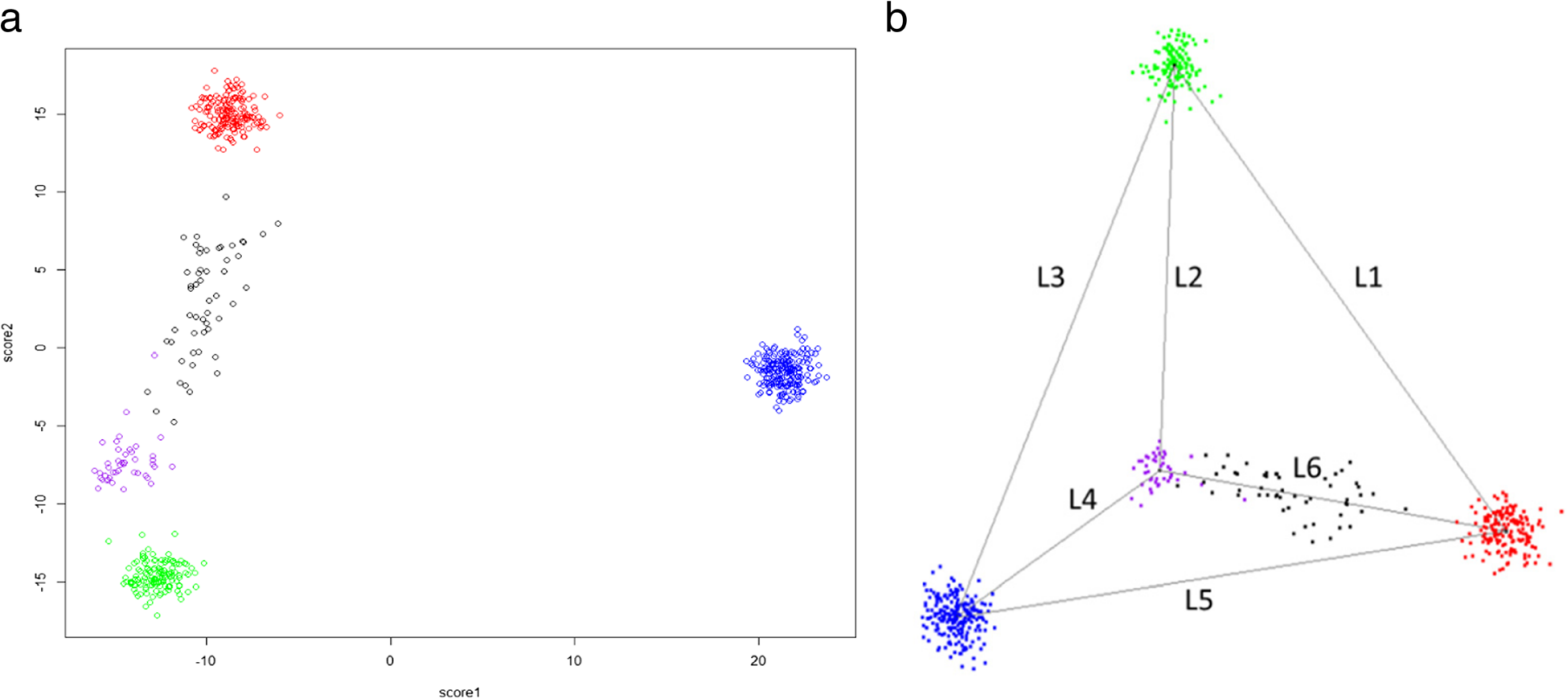
Panel **a** displays four intercontinental populations and one mixed population in 2-dimensions. Tetrahedron model in **b** can be applied to the extended intercontinental analysis. European, Asian, African, and Native American are four distinct populations and denoted in red, green, blue, purple, respectively. Mexican American is mixed population and represented in black. Each intercontinental population has three combinations derived for each face in the tetrahedron. First, FastPop is applied to infer ancestry on each face of tetrahedron and then average proportions over each intercontinental population are used to summarize ancestry

We also tested the algorithm on a data set comprising 505 individuals with European, Asian, African ancestry from the HapMap consortium along with 43 individuals of Native American ancestry derived from the Human Genome Diversity Panel, along with 53 Mexican individuals with mixed ancestry. Points at or closest to the four vertex corners were designated as 100 % European, Asian, African or Native American, respectively (red, green, blue or purple in Fig. 4). The Mexican samples had a mixed ancestry and all the points lay within the ancestry-defined tetrahedron (black color in Fig. 4). For individuals with mixed ancestry we projected the samples to the faces of the tetrahedron (Supplementary Methods). We compared the results from the extended FastPop and STRUCTURE for the four population ancestry inference, and the results were highly consistent between methods. The concordance rates were greater than 97 % in all the four populations (Additional file 1: Table S1 and Figure S1). And the correlations for the estimated proportions between FastPop and STRUCTURE were all greater than 0.99 for the four populations. The 54 Mexican samples had a mixed ancestry and they were identified as mixed population by both Structure and extended FastPop (Additional file 1: Figure S2). As noted in the methods above, individuals outside the ancestry tetrahedron are mapped to the nearest part of the tetrahedron and analyzed according to equation 4.

## Discussion

There are broadly two types of clustering methods: distance based methods and model-based methods. The algorithm of FastPop is based on distance. We first map each individual using PC values as coordinates and the joint probabilities of ancestry are based on how close each individual is to each centroid or nearest elements of ancestry surfaces. STRUCTURE is a model-based method. It assumes that each cluster (population) is modeled by a characteristic set of allele frequencies and the main modeling assumptions are Hardy-Weinberg equilibrium within populations and complete linkage equilibrium between loci within populations [2]. Structure applies a Bayesian approach to infer the ancestry of each individual and allele frequency from all populations. So FastPop and STRUCTURE have completely different algorithms although we showed that the results from FastPop are highly correlated with that from STRUCTURE in our study. FastPop is a distance-based method and it conducted mathematic calculations using distance on the coordinates which is very straightforward and fast. However, STRUCTURE uses a Bayesian method for inference of ancestry and it applies MCMC algorithm to achieve final desired distribution in computation. As shown by equations (1) and (2), calculations in FastPop do not require iteration to solve the principal components and therefore we find that FastPop works very well for moderate samples sizes such as those we have studied here and that the scoring method can then be applied effectively to any sample size. If an investigator wanted to apply our approach to a very large dataset comprising over 50,000 subjects we have found that substituting PCA with matrix inversion with a singular value decomposition method or with a random vector analysis will reduce computation time compared to application of standard PCA [9, 10].

PCA has become a standard procedure in population genetics study for substructure analysis. The eigenvectors from PCA are easy to use for population adjustment in GWA studies. However it lacks the ability to provide clear information for ancestral origin. To fill this gap, we developed an efficient tool for inference of ancestry with PCA scores as the input. The PCA scores generated by FastPop can be used to identify ancestries of individuals or could be used to adjust for population structure in association analysis. The scores are based on SNP weights so the scores of reference population such as Hapmap samples can be applied to other studies provided the same set of markers are used. This characteristic is attractive especially in large consortia studies when multiple independent studies may be analyzed by individual laboratories. The PCA scores generated in one site can be adopted by other sites thus to reduce the repetitive work and ensure consistency among analyses. In current GWA studies, sample sizes keep increasing and now involve tens of thousands individuals. FastPop can be more efficiently implement than Structure in analyzing data with a large sample size.

FastPop provides the estimated centroids from a training set considering the users may have a small data set and may require a golden standard for the centroid positions for the populations. Theoretically, the triangle model in FastPop will work without training samples. When the sample size of a data comprising of three populations is big enough, we can calculate the centroids position for each population based on the principal component values from the study samples instead of deriving centroid positions from Hapmap samples. We also tested this idea by inferring ancestry for 19661 study samples without using Hapmap samples, and the correlations of the results between FastPop and Structure were still > 95 %. For this approach, one needs to define a set of centroids for defining ancestral origins.

As a further comparative analysis, we also evaluated linear discriminative analysis (LDA) method applied to PCA scores as input to predict ancestry. Compared to LDA, FastPop had better performance in terms of the estimated proportions, consistent performance across different cut off values for decisions and a lower excess positive rate for Europeans. We are using the term ‘excess positives’ here to denote the classification of individuals who may have multiple ancestries into a single ancestry group by LDA (Additional file 1: Table S2). The improved performance of FastPop over a more generic application of LDA reflects the application of clines relating more typical intermarriages along continental clines as opposed to the more generic model that is required by LDA.

The version of FastPop released to SourceForge includes an input file with 2318 SNPs that differentiate European, African and Asian very well across the whole genome. The 2318 SNPs were derived from our study population to maximize variation among European, African and Asian populations. However, any set of markers that differentiates European, African and Asian can be applied in the analysis. We have provided a set of markers for the users so they do not need to choose a set of ancestry informative markers for the analysis. If some of the SNPs are missing from the input file, the researchers can replace the missing genotype with average of genotype from the samples we provided in the package. FastPop can be implemented for different sets of markers and the locations of three centroids would then need to be recomputed either using user supplied samples or HapMap samples with a different set of markers.

FastPop is based on a trianglular algorithm so theoretically it works for any data including different intercontinental populations provided the ancestral origins provide reasonable fit to a triangular origin. In this study, we evaluated the performance of FastPop in differentiating individuals with either a mixture of European, African and Asian or with additional Native American Ancestry. The preponderance of studies requires analysis of samples without consideration of origins, which are the three major ancestries in most genome-wide association analysis.

The currently released FastPop has been released to characterize genetic data involving three ancestries and is available upon request for four ancestries. Theoretically, our algorithm can be applied to an arbitrary number of populations, but the algorithm becomes more complex as the number of dimensions increases. We also have assumed that that the number of dimensions that need to be characterized is one less than the number of populations.

## Conclusions

We developed FastPop, an efficient R package that can be applied to ancestry study on genotype data including three intercontinental origins. The PCA scores generated by FastPop can be included for population structure adjustment or classification into major ancestral groups. Additionally, the method can be applied for large studies to ensure comparability of results among participating sites. The algorithm based on PCA score mapping can also be extended to multiple population inference. We have applied FastPop in the analysis of data from the OncoArray consortium, which has genotyped 410,000 samples, because we needed an approach that could be readily applied across this large consortium. We anticipate that our approach would be of value to other investigators performing coordinated analyses across large consortia.

## Availability and requirements

Project name: FastPop software

Project home page: https://sourceforge.net/projects/fastpop/files/

Operating system: Linux

Programming language: R

Other requirements: None

License: None

Any restrictions to use by non-academics: None

### Ethics, consent and permissions

This study has been approved by the Committee for the Protection of Human Subject (CPHS) – the Institutional Review Board (IRB) at Dartmouth College. All human subjects involved in this study consented to research involving genetic analysis. All individuals signed Institutional Review Board approved consent documents related to genetic analysis of germline samples. Samples were deidentified prior to analysis. All the analysis was performed at Dartmouth College.

## Additional file

Additional file 1: Supplementary Materials. (DOCX 35 kb)

## References

[1] Devlin B, Roeder K (1999). Genomic control for association studies. Biometrics.

[2] Pritchard JK (2000). Inference of population structure using multilocus geno-type data. Genetics.

[3] Pritchard JK (2001). Case–control studies of association in structured or admixed populations. Theor Popul Biol.

[4] Menozzi P (1978). Synthetic maps of human gene frequencies in Europeans. Science.

[5] Patterson N, Price A, Reich D (2006). Population structure and eigenanalysis. PLoS Genet.

[6] Price AL (2006). Principal components analysis corrects for stratification in genome-wide association studies. Nat Genet.

[7] Serre D, Paabo S (2004). Evidence for gradients of human genetic diversity within and among continents. Genome Res.

[8] Wang C, Zhan X, Liang L, Abecasis GR, Lin X (2015). Improved ancestry extimation for both genotyping and sequencing data using projection procrustes analysis and genotype imputation. Am J Hum Genet.

[9] Abraham G, Inouye M (2014). Fast principal component analysis of large-scale genome-wide data. PLoS ONE.

[10] Galinsky KJ, Bhatia G, Loh P, Georgiev S, Mukherjee S, et al. Fast principal components analysis reveals independent evolution of ADH1B gene in Europe and East Asia. 2015. doi: http://dx.doi.org/10.1101/018143 10.1016/j.ajhg.2015.12.022 PMC4827102 26924531

