# Supplementary material for: FastPop: a rapid principal component derived method to infer intercontinental ancestry using genetic data
Source: BMC Bioinformatics. 2016 Mar 9;17:122. doi: 10.1186/s12859-016-0965-1 (PMC4784403; doi:10.1186/s12859-016-0965-1)
Supplement: Supplementary file 1 — Supplementary Materials. (DOCX 35 kb) [file 12859_2016_965_MOESM1_ESM.docx]

**Supplementary Material**

Table 1S. Comparison of assigned ancestry using different cutoff values for FastPop and Structure for inference of four populations.

| **Cutoff value** | **CEU** | **YRI** | **CHB** | **Native American** |
| --- | --- | --- | --- | --- |
| 0.9 | 165/164/164 | 203/203/203 | 137/135/135 | 39/40/39 |
| 0.8 | 165/165/165 | 203/203/203 | 137/137/137 | 43/43/43 |
| 0.7 | 165/165/165 | 203/203/203 | 137/137/137 | 43/43/43 |

Number in each cell indicates: No. assigned by structure/No. assigned by FastPop/No. common in both methods. Structure analysis was conducted without prior population information.

Table 2S. Comparison of assigned ancestry between linear discriminative analysis (LDA) and Structure in analysis of three populations.

| **Cutoff value** | **CEU** | **YRI** | **CHB** |
| --- | --- | --- | --- |
| LDA performed using first 3 PCA scores | | | |
| 0.9 | 17520/18437/17520 | 64/363/64 | 740/858/740 |
| 0.8 | 18016/18437/18016 | 175/363/175 | 773/858/773 |
| 0.7 | 18171/18438/18171 | 266/363/266 | 799/858/799 |
| LDA performed using first 5 PCA scores | | | |
| 0.9 | 17520/18442/17520 | 64/363/63 | 740/854/740 |
| 0.8 | 18016/18442/18106 | 175/363/175 | 773/855/773 |
| 0.7 | 18171/18442/18171 | 266/363/266 | 799/855/799 |

Number in each cell indicates: No. assigned by structure/No. assigned by LDA/No. common in both methods. We first created linear discriminant functions on CEU, CHB and YRI using PCA scores from the 505 Hapmap samples with known ancestry, and then applied the functions on the PCA scores from samples with unkown origin to infer the ancestry. Posterior probability of members in each population is obtained from $Pr(pop=j|ind)=\frac{e^{-0.5\times{score}_{j}^{2}}}{\sum_{i} e^{-0.5\times{score}_{i}^{2}}}$, where i and j indicate populations. The exponential function in posterior probability calculation introduced extreme values in results and majority of the estimated proportions of ancestry were either close to 0 or 1.

We studied the use of three and five PCA scores to derive a linear model for inference. We compared the results between LDA and STRUCTURE (Tab 2S). When we used the first three PCA scores, the correlations of estimated proportions between LDA and Structure were 0.96, 0.96 and 0.95 for CEU, CHB and YRI, respectively; when used the first five PCA the correlations were 0.95, 0.96 and 0.95 for CEU, CHB and YRI, respectively. Overall, the correlations between LDA and STRUCTURE were lower than the correlations between FastPop and Structure (0.99, 0.99, and 0.97 for CEU, CHB and YRI). We also noticed that LDA introduced misclassification across the three populations in the results. For example when three PCA scores were used and 0.9 was set as the cutoff, besides the 17520 individuals with European ancestry identified by STRUCTURE, LDA identified another 917 members with European ancestry. For this setting, FastPop detected no excess positive Europeans on the same data analysis (Tab 2S for LDA and Tab 1 for FastPop). Compared to LDA, FastPop had better performance in terms of the estimated proportions, consistent performance across different cut off values for decisions and a lower excess positive rate for Europeans. We are using the term ‘excess positives’ here to denote the classification of individuals who may have multiple ancestries into a single ancestry group by LDA.


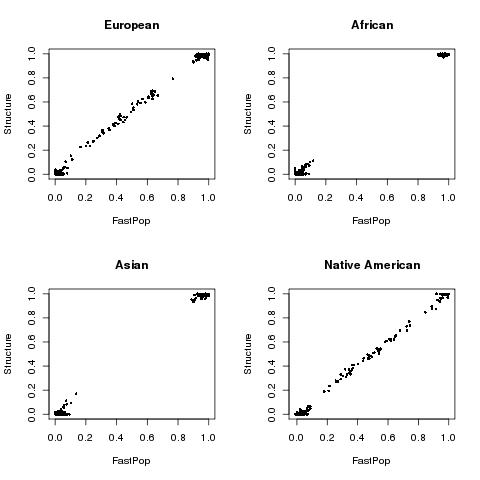


Figure 1S. Comparison of estimated proportion of ancestry between FastPop and Structure for 601 individuals coming from four origins. X and Y axes denote the proportion of ancestry for each individual from FastPop and Structure.


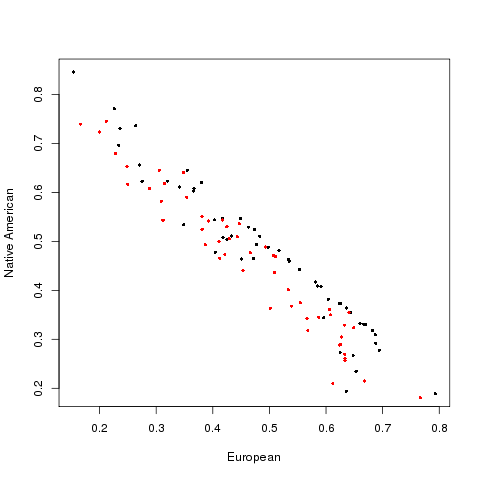


Figure 2S. Comparison of estimated proportion between Structure and FastPop for 53 Mexican individuals. Black and red colors denote results from Structure and FastPop, respectively. X and Y axes denote the proportion of ancestry for European and Native American, respectively.

Supplementary Methods projecting samples to faces of tetrahedron in a four population inference.

Consider a sample point M with coordinates(x_0_; y_0_; z_0_). Let a plane of the ancestry tetrahedron be described by the plane equation : ax+by+cz+d=0. Then the line going through M and being perpendicular to the plane is described by the system of equations : x= x_0_+at, y= y_0_+bt, z= z_0_+ct. Thus the coordinates of the point M' = (x0';y0';z0') which we require (the point projected on the plane) is at the intersection between the plane and the line perpendicular to the plane passing through M'. To find the coordinates of M' we insert the line equation into the plane equation and find the plane equation becomes :
 a(x_0_+at)+b(y_0_+bt)+c(z_0_+ct)+d=0, which can be transformed to t(a^2^+b^2^+c^2^)+ax_0_+by_0_+cz_0_+d=0. We then used the uniroot program of R to find the root of this function, which is the value of t we require. Thus we found M' and its coordinates are : x_0_'=x_0_+at, y_0_'= y_0_+bt, z_0_'= z_0_+ct.
